# A Comparative Evaluation of the Radiopacity of Contemporary Restorative CAD/CAM Blocks Using Digital Radiography Based on the Impact of Material Composition

**DOI:** 10.1155/2022/4131176

**Published:** 2022-02-21

**Authors:** Nahla Gamal Elhelbawy, Rehab F Ghouraba, Fatma A Hasaneen

**Affiliations:** ^1^Dental Biomaterials Department, Faculty of Dentistry, Tanta University, Tanta, Egypt; ^2^Oral Medicine, Periodontology, Oral Diagnosis and Radiology Department, Faculty of Dentistry, Tanta University, Tanta, Egypt; ^3^Fixed Prosthodontics Department, Faculty of Dentistry, Tanta University, Tanta, Egypt

## Abstract

**Purpose:**

The main purpose of this study was to assess the radiopacity of contemporary restorative computer-aided design (CAD)/computer-aided manufacturing (CAM) materials and the impact of material composition as measured by energy-dispersive X-ray spectrophotometry (EDX) on radiopacity.

**Materials and Methods:**

Ten specimens of six CAD/CAM materials with 1 mm thickness were produced and then digitally radiographed with an aluminum (Al) step-wedge (SW) and 1 mm thick tooth slice. The specimen mean gray values (MGVs) were recorded in pixels and compared to an Al-SW, dentin, and enamel of equal thickness. For the elementary analysis of the composition of the materials, EDX was performed.

**Results:**

The recorded MGVs ranged between 21.20 ± 4.94 and 238.5 ± 13.61 pixels. Materials were sorted according to the MGVs descendingly, Prettau, Vita Suprinity, Vita Enamic, Shofu, Pekkton, and BioHPP. Prettau and Vita Suprinity had significantly higher MGV than dentin and 1 mm thick Al. In comparison, Vita Enamic had a slightly higher value than dentin and 1 mm thick Al. Although Pekkton and BioHPP had MGV significantly lower than dentin and 1 mm thick Al, Shofu had a significantly lower value than dentin and nonsignificantly lower than 1 mm thick Al (*p* < 0.05). According to EDX analysis, the examined materials contained several components in varying quantities of radiopacity.

**Conclusions:**

The radiopacity of only three studied materials exceeded the International Organization for Standardization's minimum standards (ISO).

## 1. Introduction

Currently, the key emphasis in dentistry is aesthetics. Consequently, both patients and dentists are enthusiastic about using tooth-colored materials. For indirect dental restorations such as veneers, inlays, onlays, implant-supported crowns, bridges, and anterior and posterior crowns, CAD/CAM technology has become widespread in dental offices and laboratories [1].

Currently, there are no limits to the types of dental restorations that could be created by clinicians, as a result of the abundance and advancement of CAD/CAM technology, systems, milling machines, and other tools. Composite resin, leucite-reinforced, lithium-disilicate glass ceramics, hybrid ceramics (polymer-based), and polycrystalline ceramics (zirconia) are among the systems and block materials developed by the manufacturer [2]. Due to various CAD/CAM blocks, adequate strength requirements, esthetic aspirations, treatment time intervals, and accuracy, dentists face a challenge in selecting the suitable restorative material [3]. Recently, radiopacity has become a popular property in high-quality CAD/CAM materials.

The radiopacity controls the degree of material reflection and offers a suitable contrast to the tooth structure on a radiograph. Therefore, it is a critical demand for restorative materials. Consequently, the Council on Dental Materials, Instruments, and Equipment updated the specifications for resin-based restorative materials, adding radiopacity to the biological, physical, and mechanical criteria [4]. Ceramic material radiopacity enables the radiological detection of restoration forms, contours, and defects, which enhances the diagnosis of recurrent caries under restoration. It allows for the study of the periodontal effects of overhangs, making them a valuable diagnostic aid for determining the long-term durability of restorations [5–7]. Furthermore, it aids in locating fixed or removable dental prostheses and temporary crowns swallowed accidentally by patients [8]. As a result, assessing the radiopacity values of CAD/CAM materials has a substantial impact on the best restorative block selection.

There are two common approaches for determining the radiopacity of dental materials: the conventional method using transmission densitometry and digital image analysis (digital radiography). Digital technology includes two types: direct and indirect. The optical density value is acquired directly using digital image analysis. The radiopacity of a material could be quantified on a scale of 0–255 using a software program with the direct digital approach [9]. Direct digital systems have the key benefits of immediate image acquisition, the absence of processing chemicals, a broad dynamic range, and enhanced radiation sensitivity. The digital system phosphor storage plate (PSP) has been verified to be safe, rapid, and user-friendly [10].

Radiopacity of the materials is commonly assessed by comparing them to enamel, dentin, or Al. Some studies have indicated that restorative materials should have a radiopacity equal to or greater than dentin [11, 12]. In contrast, others have suggested that restorative materials should have radiopacity values equal to or greater than enamel [13, 14]. Dentin radiopacity was proven to be relatively similar to that of Al of the same thickness, whereas enamel radiopacity was proven to be nearly double to that of Al at the same thickness [15, 16]. The radiopacity of a dental material is noted as an optical density value or in terms of equivalent (Al) thickness (in millimeters) for comparison with the other research studies. In addition, it should be equal to or greater than that of the same thickness of Al, according to the (ISO) 4049 : 2009 specification [17, 18]. Furthermore, it should be no less than 0.5 mm of any manufacturer's specified value [19].

Companies are launching fully stabilized zirconia (FSZ) as a more translucent material. Prettau anterior is an example of a translucent monolithic FSZ [20]. Vita Suprinity is a ceramic made of lithium silicate reinforced with zirconium dioxide (ZLS) and marketed a few years ago to achieve the desired esthetic and strength characteristics [21]. Resin-matrix ceramics (RMCs) have a higher load capacity, improved modulus of elasticity, and superior milling quality than silica-based ceramics [22]. Vita Enamic is an example of hybrid ceramics (HC) with a ceramic network infiltrated with a polymer. Shofu HC block is a resin-based ceramic that contains a polymer matrix with at least 80% nanosized ceramic filler particles.

Polyetheretherketone (PEEK) and polyetherketoneketone (PEKK), which belong to the superordinate group of polyaryletherketone (PAEK), have been extensively recommended for use in fixed prosthodontics recently. With the rapid advancement of CAD/CAM technology, high-performance polymers have emerged as substitutes to metal and glass ceramics for prosthetic restorations [23, 24].

There are limited publications for the radiopacity of contemporary restorative CAD/CAM materials. Consequently, the purpose of this research was to evaluate the radiopacity of six types of existing and new CAD/CAM restorative materials using a digital image analysis method and then to compare the radiopacity values of these materials with those of enamel, dentin, and Al-SW of varying thicknesses. In addition, the constituent elements of each type of CAD/CAM material were examined by EDX to determine which elements have an impact on radiopacity.

## 2. Materials and Methods

### 2.1. Specimen Preparation

The radiopacity of six CAD/CAM restorative block materials was assessed using digital radiography.  Table 1 provides each category of materials, manufacturers, trade names, and chemical components. Using a low-speed diamond saw (Isomet, Buehler, Lake Bluff, IL, USA) with a blade speed of 2500 rpm and a feed rate of 14.7 mm/min under water cooling, a total of sixty disc specimens with a 10 mm diameter and 1 mm thickness were produced from every material block (*n* = 10 for each type). Complete sintering of Prettau® anterior samples was performed following the manufacturer's instructions in a furnace (Zirkonofen 600/V2, Zirkonzahn, Taufers, Italy). Crystallization of Vita Suprinity discs was carried out in (EP 3010 Programat, Ivoclar Vivadent, Schaan, Liechtenstein) a furnace following the manufacturer's guidelines. The discs were smoothed with wet #400, #800, and #1200 grit silicon carbide paper. Using digital calipers (Electronic Digital Caliper, Shan, China), every specimen thickness was affirmed as 1 mm (±0.01 mm). Subsequently, they were ultrasonically washed in distilled water for 10 minutes. Afterward, all discs were kept wet at 37°C until the radiography stage of the experiment was performed.

The specimens were divided into the following six groups (*n* = 10 for each group) based on the type of material:

- Group 1: fully stabilized zirconia (FSZ) (Prettau anterior).
- Group 2: zirconia-containing lithium silicate ceramics (ZLS) (Vita Suprinity).
-   Group 3: hybrid ceramics (HC) (Vita Enamic).
- Group 4: resin-based ceramics (RBC) (Shofu).
- Group 5: high-performance polymer polyetherketoneketone (PEKK) (Pekkton).
- Group 6: high-performance polymer polyetheretherketone (PEEK) (breCAM.BioHPP).

The Ethics Committee of Tanta University Faculty of Dentistry gave approval for this study. Specimens of enamel and dentin were obtained by inserting an extracted human molar free of caries in acrylic resin, cutting it in a transverse direction to make a 1 mm thick piece in the same manner as the specimen, and then keeping it in purified water at 37°C waiting to be utilized.

### 2.2. Radiographic Analysis

The radiopacity of each CAD/CAM restorative material was compared with Al thickness and verified for compliance with the (ISO/4049) standards for restorative material radiopacity using a graduated 99.5% pure Al-SW having sixteen incremental steps with a thickness of 1 mm and a length of 2 mm (1–16 mm) as an internal standard for each radiography exposure [17].

**Figure 1:**
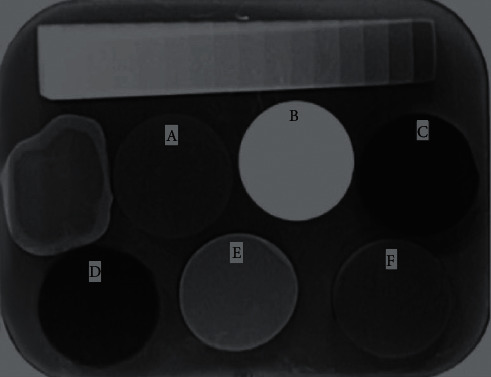
Digital radiograph of each CAD/CAM restorative material specimen, tooth structure slice, and aluminum step-wedge. (a) Shofu. (b) Prettau. (c) BioHPP. (d) Pekkton. (e) Vita Suprinity. (f) Vita Enamic.

All samples were deposited directly onto the intraoral digital phosphor plate (PSP) sensor (Apixia HD, Digital Dental Ltd. Westhoughton, Bolton, UK), in addition to a 1 mm thick tooth slice and Al-SW. The X-ray apparatus (New Life Radiology S.R.L, Grugliasco, Torino, Italy) was adjusted to 70 kVp, 7 mA, 0.6 s exposure time, focal spot of 0.7 mm, and a focus-sensor distance of 30 cm for radiographic investigations, and the central X-ray beam was directed at a 90° angle. The digital radiography technique was repeated by an oral radiologist ten times with the same exposure conditions each time (Figure 1). A zone of interest was identified on the digital radiography image in the center of each test material, on the dentin and enamel of the tooth slice, and in each step of Al-SW. A software application (APIXIA® PSP Digital Imaging Software) was used to determine the dentin and enamel of the tooth slice, each step of the Al-SW, and the mean gray values of the sample (MGV). To avoid discrepancies in the results due to radiography methods, the calculation of MGVs was performed three times. Thereafter, the MGVs of the investigated materials were compared with those of enamel, dentin, and Al-SW of equal thickness. Last, all radiographs were assessed by a single oral radiologist.

### 2.3. Measurements of Elemental Composition via Energy-Dispersive X-Ray (EDX)

**Figure 2:**
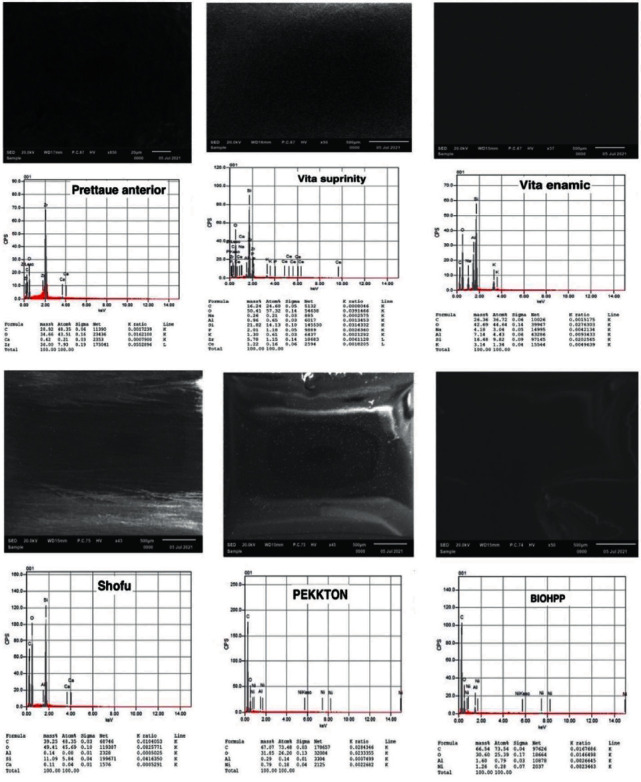
Representative EDX spectrum of the surface of CAD/CAM restorative materials specimens.

The morphology and chemical contents of the CAD/CAM restorative material disc were examined using scanning electron microscopy coupled with EDX (JEOL, Japan JSM-IT 100). Each specimen was removed from the packaging with sterile forceps and mounted onto the sample holder without touching the surface. Before closing the chamber, the specimens were cleaned with ethyl alcohol to eliminate any material artifacts such as dust. After that, a vacuum was created, and imaging and measurements were carried out. EDX analysis was performed using the JEOL software (JEOL, Japan, JSM-IT 100) on areas of equal distance from the center of each CAD/CAM restorative material as shown in Figure 2.

### 2.4. Statistical Analysis

The normal distribution test was used to examine the distribution of MGVs data for normality. There was a normal distribution in the data. One-way analysis of variance (ANOVA) was used to compare the differences between the MGVs of all groups. Moreover, Tukey's post hoc test was used to examine the differences between CAD/CAM restorative materials. The MGVs were compared using a one-sample *t*-test. SPSS version 11.0 was used to analyze the data (SPSS Inc., Chicago, IL, USA), and significance was established at a *P* value of <0.05.

## 3. Results

The MGVs of all CAD/CAM restorative materials were compared, as given in Table 2. Prettau, Vita Suprinity, Vita Enamic, Shofu, Pekkton, and BioHPP were ranked according to MGVs in descending order. BioHPP had the lowest value of 21.20 ± 4.940 pixels among the groups, while Prettau had the highest value of 238.5 ± 13.61 pixels. Furthermore, the Tukey multiple comparison test reported a significant difference (*p* < 0.05) between the tested materials, except between Shofu and Pekkton (*p* > 0.05).

**Figure 3:**
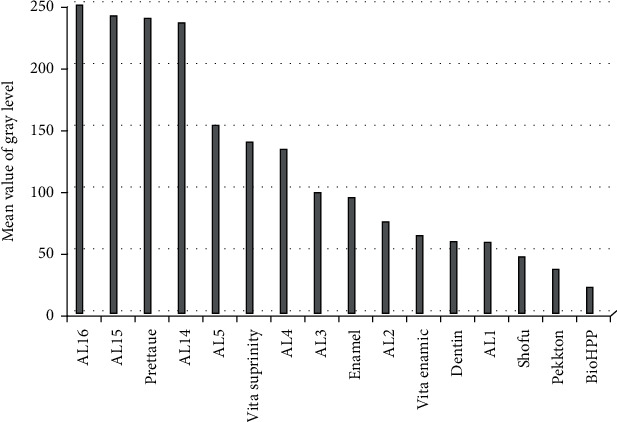
MGVs in pixels of tested CAD/CAM restorative materials compared to enamel, dentin, and Al-SW.

Table 2 and Figure 3 provide the comparison results for all MGVs of materials with enamel, dentin, and Al-SW. According to the paired-sample *t*-test, Prettau and Vita Suprinity exhibited MGVs that were significantly higher than dentin and 1 mm thick Al. Vita Enamic had a nonsignificantly higher value than both dentin and 1 mm thick Al. On the contrary, Pekkton and BioHPP had MGVs that were significantly lower than dentin and 1 mm thick Al. In comparison, Shofu had significantly lower values than dentin and nonsignificantly lower than 1 mm thick Al (*p* < 0.05).

Prettau and Vita Suprinity had significantly greater MGVs than enamel, while the remaining groups had significantly lower values (*p* < 0.05) (Table 2 and Figure 3). Shofu and Vita Enamic MGVs did not differ significantly from 1 mm thick AL, whereas Prettau MGVs did not differ significantly from 14 mm thick AL. Vita Suprinity did not differ significantly from that of 4 mm thick AL. On the contrary, Pekkton and BioHPP exhibited significantly lower values than 1 mm thick AL.

Table 3 provides the radiopaque elements and their mean atomic percentages obtained by the elementary analysis of EDX. Based on the results obtained, it was revealed that each manufacturer utilized different elements to achieve the required radiopacity of the tested materials. The examined CAD/CAM materials contained zirconium, aluminum, and silicon in various ratios for attaining radiopacity. Prettau had the greatest MGVs and contained zirconium (16.34% ± 1.38), whereas Pekkton and BioHPP had the lowest MGVs and had Al (0.85% ± 0.07) and (0.22% ± 0.07), respectively.

## 4. Discussion

Radiopacity, biocompatibility, adhesion, and esthetic are the principal aspects that should be considered when selecting suitable materials by clinicians. The radiopacity of a material is recognized as the inverse of its optical density in a radiographic image [25]. Radiographic images of the materials studied were taken in the current investigation utilizing the digital radiography system using a storage phosphor plate. This system was chosen to achieve precise and reliable optimal gray pixel values for radiopacity, as well as to provide a more consistent image that may eliminate the need for an Al-SW if the film-target distance and exposure variables were kept constant [26, 27]. EDX analysis was used to investigate the impact of material composition on radiopacity.

In the present study, the radiopacity of dentin results was close to 1 mm Al, and the radiopacity of enamel was nearly twice that of dentin, which agreed with the study of Yasa et al. 2015 and Atala et al. 2019 [15, 16]. The radiopacities of the CAD/CAM restorative materials investigated varied significantly, except for Shofu and Pekkton. This finding revealed that the type of material studied and its constituents, such as glass, ceramic, and/or resin, as well as metal filler particles such as zirconium and aluminum, affected radiopacity, which was in line with the studies of Atala et al. 2019, Hara et al. 2001, and Wiesli, Özcan 2015 that have verified this diversity in radiopacity in their results [16, 28, 29]. The highest radiopacity was found in Prettau while the lowest was in BioHPP. This finding is attributed to Prettau being strengthened with (16.34% ± 1.38) zirconium, while BioHPP has (0.22% ± 0.07) aluminum as a metal filler, as revealed by the EDX analysis.

The MGV results of Pekkton and BioHPP were significantly lower than dentin and the same thickness of AL-SW due to the percentage of radiopaque elements in them that were Al (0.85% ± 0.07) and Al (0.22% ± 0.07), respectively. At the same time, Shofu had significantly lower MGVs than dentin and slightly lower values than the same thickness of AL-SW. The lower Shofu results were due to the presence of Al (0.14% ± 0.1) and Si (5.43% ± 0.39), which was agreed with Atala et al. 2018 who linked the lower radiopacities of hybrid blocks (Shofu) with the dense glass matrix in their contents and Koizumi et al. 2020 who related their results to the absence of barium [16, 29]. Therefore, we suggested adding heavy metals such as barium, strontium, and zirconium to Shofu, Pekkton, and BioHPP to increase radiopacity, which was consistent with the suggestions of Wiesli and Özcan 2015, Rauchrt and others 2020, and Koizumi et al. 2020 [29–31]. Because using materials with lower-than-accepted MGVs may impede the determination of the faulty proximal contour, as well as the diagnosis of recurrent caries and other flaws that can contribute to clinical failure [13]. In addition, they will rely on the radiopacity of the luting materials for the detection of recurrent caries under restoration.

Three of the six materials tested exhibited higher MGVs than dentin (Prettau, Vita Suprinity, and Vita Enamic) which met ISO Standard 4049. In addition, both Prettau and Vita Suprinity outperform enamel. This higher value was due to the presence of zirconium, aluminum, and silicon. The high MGVs observed by Prettau agreed with Pekkan et al. 2016 who reported superior radiopacity of Y-TZP ceramics, probably as a result of the high atomic number and molecular weight of yttrium and zirconium [32]. Vita Suprinity results were compatible with those obtained by Atala et al. 2018, who linked a higher radiopacity value to high zirconium composition. However, Vita Enamic results contradicted the results of Atala et al. 2018 and Koizumi et al. 2020, who found a lower radiopacity value than dentin [16, 29]. These discrepancies in results are attributed to the radiography systems used during the study.

Although the superior limit of radiopacity has not been defined, some authors believe it should exist because very radiopaque materials make it difficult to detect marginal adaptation, recurrent caries, and other flaws on radiographs [13]. Excessive radiopacity can also generate the Mach effect, which is a visual illusion that darkens the dark border area by enhancing the contrast between two regions of different radiopacities [32, 33]. In consistent with Gama et al. 2020, the radiopacity of zirconia was the highest, and zirconia restorations may create artifacts in X-ray examination [34].

## 5. Conclusions

According to the results obtained from this study, it can be concluded that the evaluated restorative CAD/CAM materials had significantly varying radiopacity values, as each manufacturer used distinct proportions of components such as zirconium, aluminum, and silicon to achieve radiopacity. The radiopacity of Prettau, Vita Suprinity, and Vita Enamic blocks exceeded ISO minimum guidelines. In contrast, Shofu, Pekkton, and BioHPP blocks had lower radiopacity values than dentin.

Clinical significance: comprehensive knowledge of the radiopacity of materials allows clinicians to select the appropriate material to achieve clinical success. It is a useful diagnostic aid for determining the long-term durability of restorations.

## Figures and Tables

**Table 1:** Detailed description of materials tested in the study.

Material description	Manufacturer	Trade name	Chemical composition
Fully stabilized zirconia (FSZ)	Zirkonzahn, Taufers, Italy	Prettau anterior	<12% Y_2_O_3_, <1% Al_2_O_3_, max., 0.02% SiO_2_, max., 0.01% Fe_2_O_3_, max. 0.04% Na_2_O.
Zirconia-containing lithium silicate ceramics (ZLS)	Vita Zahnfabrik, Bad Säckingen, Germany	Vita Suprinity	56–64% SiO_2,_ 15–21% Li_2_O, 8–12% ZrO_2_, 1–4% K_2_O, 3–8% P_2_O5, 1–4% Al_2_O_3_, and pigments (0–6%)
Hybrid ceramics (HC)	Vita Zahnfabrik, Bad Säckingen, Germany	Vita Enamic	Ceramic part: 86% wt. SiO_2_ (58–63%), Al_2_O_3_ (20–23%), Na_2_O (9–11%), K_2_O (4–6%), B_2_O_3_ (0.5–2%), ZrO_2_ (<1%), KaO (<1%).SiO_2_ (58–63%), Al_2_O_3_ (20–23%), Na_2_O (9–11%), K_2_O (4–6%), B_2_O_3_ (0.5–2%), ZrO_2_ (<1%), KaO (<1%). Polymer part: 14 % wt (UDMA, TEGDMA)
Resin-based ceramics (RBC)	Shofu Inc., Kyoto, Japan.	Shofu disk HC	Silica powder, zirconium silicate, UDMA, TEGDMA, micro-fumed silica, silica (20 nm), barium glass (300 nm), and pigments.
High-performance polymer (HPP-PEKK)	Cendres + Métaux Italia s.r.l. Milano, Italy	Pekkton® ivory	Polyetherketoneketone (PEKK), titanium dioxide pigments.
High-performance polymer (HPP-PEEK)	Bredent, GmbH and Co.KG. Weissenhorner, Senden, Germany	breCAM.BioHPP	Partially crystalline polyetheretherketone (PEEK), 20% wt, ceramic fillers (0.3–0.5 *µ*m)

**Table 2:** MGVs in pixels (mean ± standard deviation) and statistical differences between different CAD/CAM restorative materials (for each group *n* = 10).

Groups	Mean ± SD	Minimum	Maximum	Enamel (mean = 93.60)	Dentin (mean = 58.10)	AL1 (mean = 57.10)	AL2 (mean = 74.00)	AL3 (mean = 97.70)	AL4 (mean = 132.3)	AL5 (mean = 152.0)	AL14 (mean = 234.9)	AL15 (mean = 240.4)	AL16 (mean = 249.6)
Prettau	238.5 ± 13.61^b^	213	254	S	S	S	S	S	S	S	NS	NS	NS
Vita Suprinity	138.3 ± 11.27^d^	117	155	S	S	S	S	S	NS	S	S	S	S
Vita Enamic	63 ± 10.73^e^	46	78	S	NS	NS	S	S	S	S	S	S	S
Shofu	45.50 ± 11.40^a^	34	69	S	S	NS	S	S	S	S	S	S	S
Pekkton	35.50 ± 4.428^a^	29	43	S	S	S	S	S	S	S	S	S	S
BioHPP	21.20 ± 4.940^c^	14	29	S	S	S	S	S	S	S	S	S	S

The MGVs of all the materials tested, enamel, dentin, and Al step-wedge were compared by a paired sample *t*-test. Different letters among tested groups indicate significant differences (*p* < 0.05). NS, nonsignificant (*p*<0.05); S, significant (*p*<0.05).

**Table 3:** The compositional analysis with EDX of CAD/CAM restorative materials (mean atomic % values ± standard deviations).

Materials	Composition	Radiopaque elements (mean atomic % ± standard deviation)
Prettau	C, O, Ca, Zr	Zr (16.34% ± 1.38)
Vita Suprinity	C, O, Na, Al, Si, P, K, Zr, Ce	Al (0.81% ± 0.12), Si (14.24% ± 0.74), Zr (1.47% ± 0.32)
Vita Enamic	C, O, Na, Al, Si, K	Al (4.82% ± 0.35), Si (10.49% ± 0.51)
Shofu	C, O, Al, Si, Ca	Al (0.14% ± 0.1), Si (5.43% ± 0.39)
Pekkton	C, O, Al, Ni	Al (0.85% ± 0.07)
BioHPP	C, O, Al, Ni	Al (0.22% ± 0.07)

## Data Availability

The data used to support the findings of this study are included within the article and are available from the corresponding author upon request.
